# Intravenous methadone causes acute toxic and delayed inflammatory encephalopathy with persistent neurocognitive impairments

**DOI:** 10.1186/s12883-021-02108-9

**Published:** 2021-02-22

**Authors:** Jonathan Repple, Svea Haessner, Andreas Johnen, Nils C. Landmeyer, Andreas Schulte-Mecklenbeck, Marc Pawlitzki, Heinz Wiendl, Gerd Meyer zu Hörste

**Affiliations:** 1 Department of Neurology with Institute of Translational Neurology, University Hospital Muenster, Albert-Schweitzer-Campus 1, A1, 48149 Münster, Germany; 2 Department of Psychiatry, University of Münster, Münster, Germany

**Keywords:** Methadone, Encephalopathy, Cognition, Flow cytometry, Case report

## Abstract

**Background:**

The mu-opioid agonist methadone is administered orally and used in opioid detoxification and in the treatment of moderate-to-severe pain. Acute oral methadone–use and –abuse have been associated with inflammatory and toxic central nervous system (CNS) damage in some cases and cognitive deficits can develop in long-term methadone users. In contrast, reports of intravenous methadone adverse effects are rare.

**Case presentation:**

Here, we report a patient who developed acute bilateral hearing loss, ataxia and paraparesis subsequently to intravenous methadone-abuse. While the patient gradually recovered from these deficits, widespread magnetic resonance imaging changes progressed and delayed-onset encephalopathy with signs of cortical dysfunction persisted. This was associated with changes in the composition of monocyte and natural killer cell subsets in the cerebrospinal fluid.

**Conclusion:**

This case suggests a potential bi-phasic primary toxic and secondary inflammatory CNS damage induced by intravenous methadone.

**Supplementary Information:**

The online version contains supplementary material available at 10.1186/s12883-021-02108-9.

## Background

The mu-opioid agonist methadone is administered orally and is widely used in opioid detoxification and maintenance programs and in the treatment of moderate-to-severe pain in some countries. Oral methadone–use and –abuse have been associated with potential inflammatory and toxic central nervous system (CNS) adverse effects such as cerebellitis [1] and delayed-onset toxic leukoencephalopathy [2–4]. Moreover, cognitive deficits have been reported in long-term methadone users [5]. However, case reports of intravenous methadone application [6] are rare and limited to ischemic changes or hypoxia-induced encephalopathy.

Here, we report a 19-year old male patient who subsequently to recreational intravenous methadone-abuse initially presented with acute neurological deficits with magnetic resonance imaging changes in the bilateral basal ganglia and the cerebellum. Subsequently, the patient developed wide-spread white matter T2-hyperintense lesions and delayed-onset encephalopathy accompanied by signs of secondary inflammation. Cerebral spinal fluid (CSF) flow cytometry revealed an inversion of monocyte and natural killer cell subsets while extensive neuropsychological examination demonstrated persistent cognitive deficits. This suggests a potential sequential toxic and secondary inflammatory CNS damage induced by intravenous methadone.

## Case presentation

A 19-year old Caucasian male presented to the University Hospital of Muenster emergency department after being unresponsive for 5 h. After consuming liquid methadone intravenously the night before, the patient awoke and immediately complained of partial bilateral hearing loss, walking impairment and numbness of both inner thighs.

According to third-party history obtained from two accompanying friends (the patient was amnestic regarding the night before) the patient had consumed 3 × 2.5 mL of liquid methadone (produced for substitution therapy) intravenously. They reported observing unconsciousness, urinary incontinence, and a twist of his eyes. The patient and accompanying friends admitted to occasionally orally using illegally obtained methadone and cannabis but credibly denied consuming these substances or other opiods and illegal drugs in the days before. This was the first event of intravenous methadone abuse. The medical history of the patient was otherwise unremarkable.

In the neurological examination the patient presented fluctuating consciousness, severe psychomotor and cognitive slowing (slowed speech, increased response latency, concentration deficits), mild paraparesis of the lower limbs, clonus when testing the left patellar reflex and symmetrical bilateral hypoesthesia of the inner thigh. Medical examination showed a heart rate of 76 beats per minute, blood pressure 121/73 mmHg, peripheral oxygen saturation of 97% and temperature of 36.8 °C. An electrocardiogram (ECG) showed only unspecific change of the ST-segment in V2 and V3. FAST-ultrasound did not reveal any abnormalities.

**Fig. 1:**
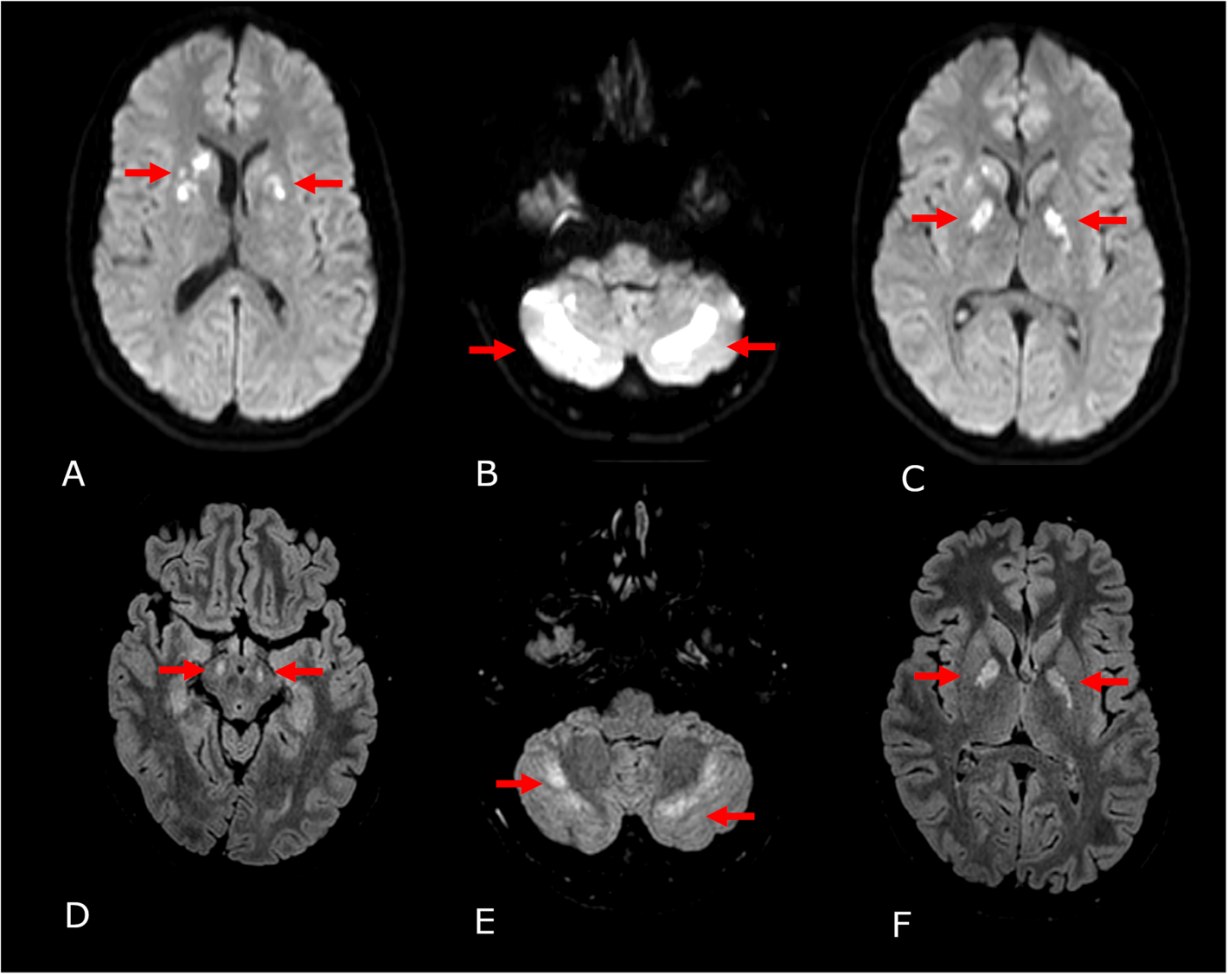
MRI imaging on day 1 and day 11 after intoxication. Timepoint 1 (Day 1 after intoxication) DWI sequences: **a** 1 basal ganglia lesions; **b** Bilateral cerebellar lesions; **c** Capsula interna lesions, posterior limb. Timepoint 2 (Day 11 after intoxication) FLAIR sequences: **d** new lesion at timepoint 2 in crura cerebri; **e** cerebellar lesions; **f** Capsula interna lesions, posterior limb

Blood tests revealed a slightly elevated c-reactive protein (5.2 mg/dl; reference < .5 mg/dl), an elevated GOP (400 U /l; reference: < 30 U/l), GPT (118 U/l; reference < 40 U /l) and an increased creatin-kinase (7104 U/l; reference: < 174 U/l) and lactatdehydrogenase (563 U/l; reference 117–217 U/l). Sodium and potassium levels were within normal range. Intoxication screening of the urine was positive for methadone (2.55 mg/l) and cannabinoids (THC-COOH: 29 μg/l) but was negative for other drugs including non-methadone opioids and barbiturates, benzodiazepines, tricyclic antidepressive drugs, methamphetamines, cocaine, phencyclidine and paracetamol. An initial magnetic resonance imaging (MRI) showed multifocal, bilateral edema of the basal ganglia (Fig. 1a), of both cerebellar hemispheres (Fig. 1b) as well as the capsula interna (Fig. 1c) with diffusion restriction and apparent diffusion coefficient (ADC) signal reductions and fluid attenuated inversion recovery (FLAIR) imaging revealed hyperintense alterations in those areas. In addition, DWI and FLAIR imaging presented mild, confluent white matter abnormalities above the lateral ventricle (Supplementary figure 1A, B). A time-of-flight (TOF)-angiography was normal. An initial spinal tap and subsequent cerebrospinal fluid analysis revealed a disturbance of the blood-brain barrier but normal total protein (542 mg/l) and normal lymphocyte counts (4/μL) and no intrathecal antibody synthesis. An electroencephalogram (EEG) revealed intermittent deceleration without epileptic discharges.

After admission, the patient was continuously awake and responsive and did not require intensive care treatment. Initially, walking was impaired without a need for a walking aid.

A transesophageal echocardiography revealed no cardiac abnormalities and especially no endocarditis. Repeated blood cultures were negative. Ear-nose-throat consultation revealed an injury of the inner ear with a diminished ability to hear below 55 dB. We performed treatment with prednisolone for 3 days (days 7–9) at 1 mg per kilogram body weight, which did not cause any immediate conceivable improvement of hearing but the hearing ability improved continuously. The unsteady gait improved to an almost normal level between days 9–11 of inpatient treatment. In contrast, cognitive deficits remained unchanged with increased response latency, slowed speech and concentration deficits. Additionally, the hypoesthesia of the inner thigh remained unchanged.

**Fig. 2:**
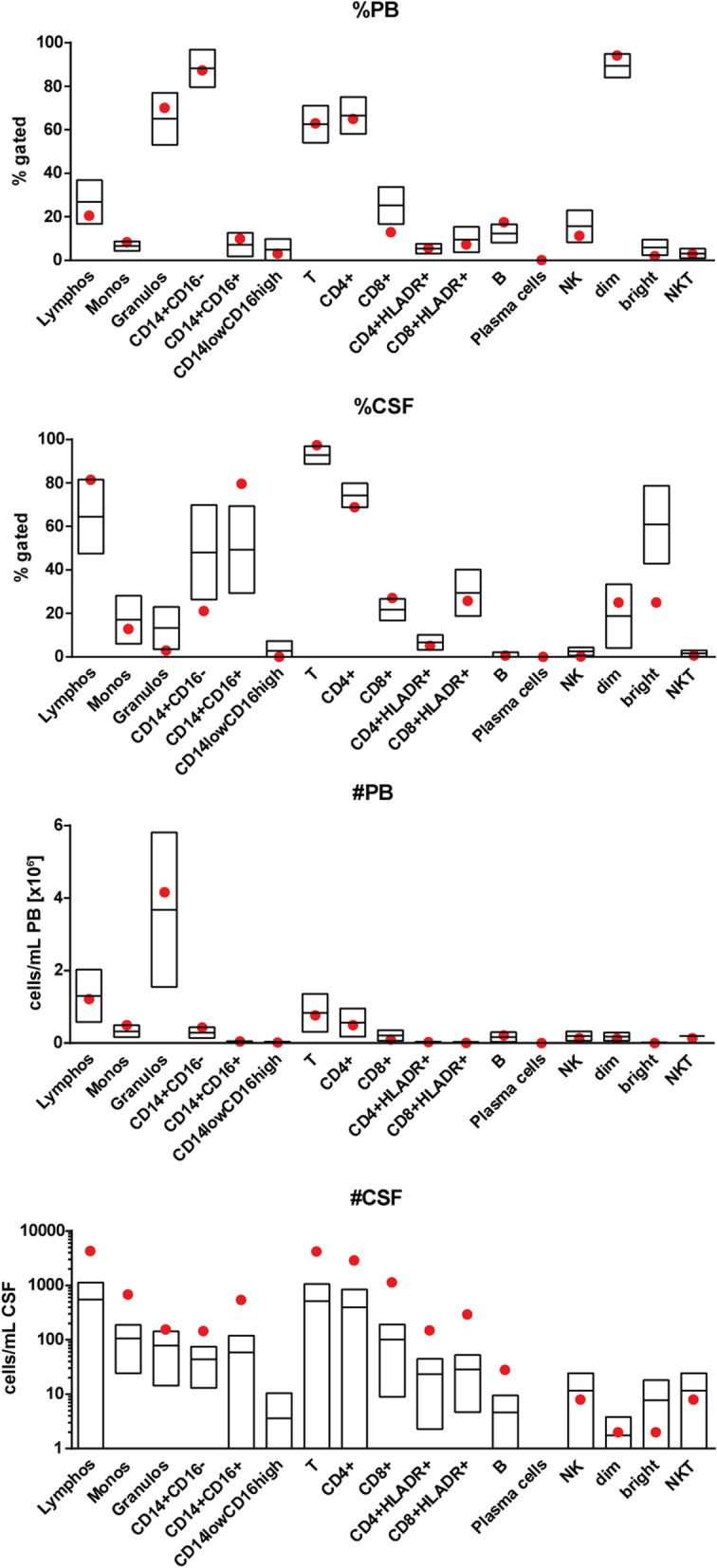
Flow Cytometry on day 11 after intoxication. Red dots indicating values of the patients, boxplot based on 29 patients with somatoform disorder (mean and 2 SD). 1: Gated cerebral spinal fluid (CSF) results. 2: Gated peripheral blood (BP) results. 3: Total CSF results. 4: Gated CSF results

An additional MRI after 11 days revealed the known FLAIR-hyperintense lesions of basal ganglia, capsula interna (Fig. 1f) and subtle abnormalities above the lateral ventricles (Supplementary figure 1C, D) as well as the cerebellar hemispheres (Fig. 1e), which were now ADC increased. Interestingly, new lesions were found in the crura cerebri bilaterally (Fig. 1d). Another spinal tap revealed an increase of lymphocytes (11/μl), but otherwise no pathological findings with a normal blood-brain barrier. Flow cytometry analysis of cerebrospinal fluid (CSF) cells revealed a shift in monocyte subtypes with a significant increase of the non-classical CD14 + CD16+ monocyte-fraction and decrease of the CD56bright natural killer cell-fraction in the lymphocyte subset in the CSF (Fig. 2). Reference values had been previously collected from 29 patients (female: 58%, mean age: 24.1y + − 5.0 standard deviation (SD)) with psychsosomatic disorder (exclusion of inflammatory CNS disorder; CSF: less than 5 cells/μl, normal protein, no intrathecal antibody synthesis).

With improved walking and hearing but considerable neurocognitive impairment we discharged the patient after 13 days to subsequent rehabilitation.

**Fig. 3:**
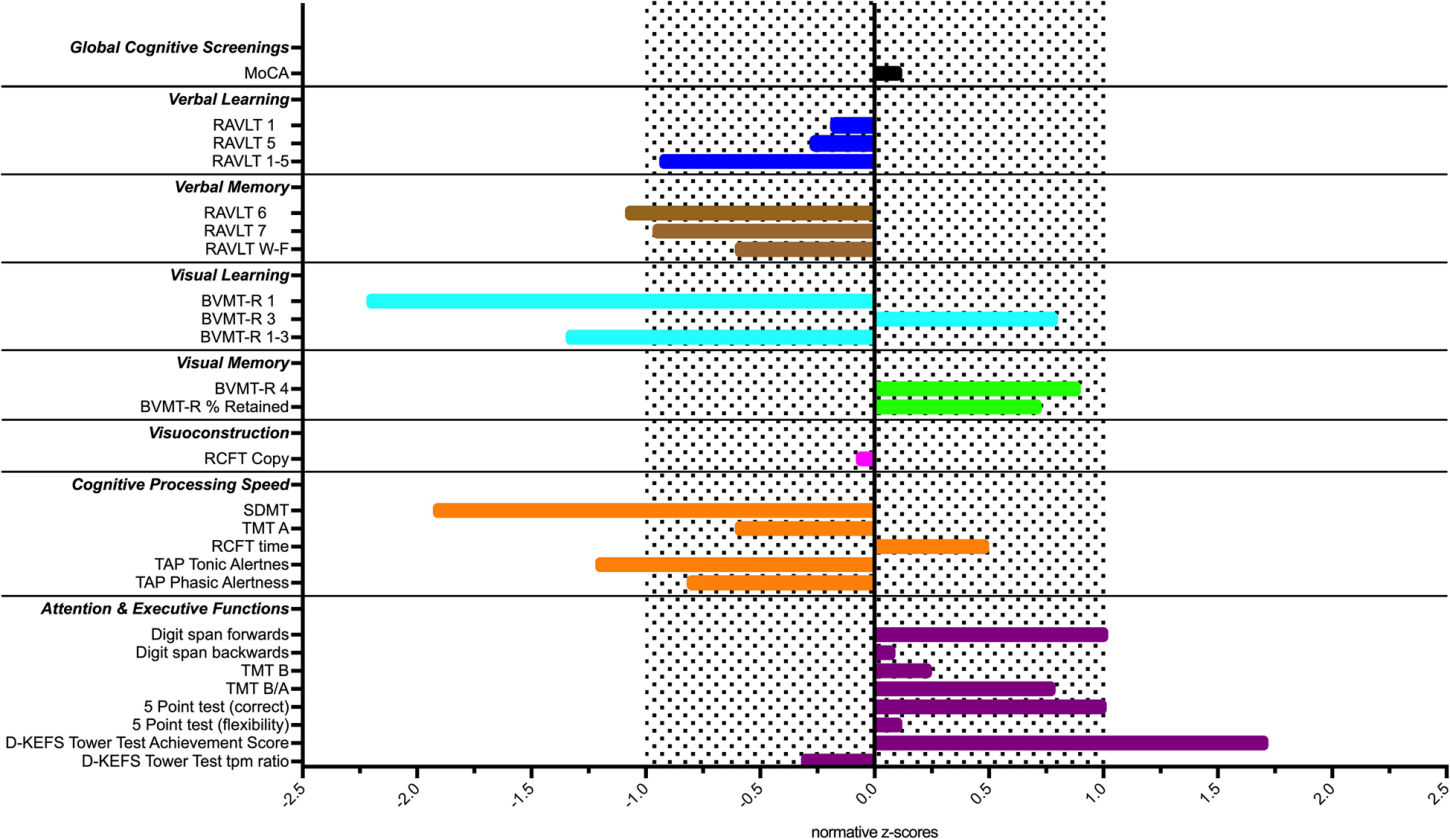
Results of the neuropsychological assessment on day 18 after intoxication. Test parameters are depicted as *z*-scores stratified for age and education. Negative values indicate worse performance compared to the normative sample. Performances within the dotted area are within 1 SD from the mean of the normative sample (z = 0 +/− 1). Test parameters are grouped into broader cognitive domains (in bold and italics). Mild-to-moderate deficits in tests for cognitive processing speed, verbal learning and memory as well as efficiency of visual learning were observed. MoCA = Montreal Cognitive Assessment; RAVLT = Rey Auditory Verbal Learning Test; BVMT-R = Brief Visual Memory Test Revised; RCFT = Rey Complex Figure Test; SDMT = Symbol Digit Modalities Test; TMT = Trail Making Test (Part A and B); TAP = Test for Attentional Performance; D-KEFS=Delis-Kaplan Executive Functions System

A neuropsychological assessment on day 18 revealed mild-to-moderate overall cognitive impairments when compared with normative data stratified for age and education (Fig. 3, Supplementary Table S1 for raw scores). Particularly tests assessing cognitive processing speed (e.g., TAP, SDMT, TMT, Time to copy a complex figure) showed consistent alterations from the norm. Learning efficiency of both verbal (RAVLT) and visual material (BVMT-R) was also impaired whereas recall from memory was only reduced for verbal but preserved for complex visual material. Interestingly, the patient showed preserved performance in tests for complex attention and higher executive functions such as planning abilities (D-KEFS Tower Test).

## Discussion and conclusions

Here we report a case of intravenous methadone-induced CNS damage that is unusually bi-phasic in presentation when compared to the available literature. The initial stage was characterized by acute-onset focal neurological signs and localized signs of diffuse CNS toxicity including ischemic changes in MRI while the second stage showed a gradual progression of more widespread damage and cognitive deficits with signs of local immune activation. We thus speculate that the initial damage may have triggered secondary intrathecal inflammation.

Compared to previous case reports, which described oral consumption of methadone ranging from 1,28 mg/L [6] to 0,21 mg/L [7], the present patient consumed methadone at a considerably higher dose. This could explain why acute onset neurological changes have been rarely described while delayed onset of methadone-induced encephalopathy is well known [3]. Our patient also consumed methadone not orally but intravenously. Previous reports describe oral ingestion of methadone or oral or inhaled heroine leading to symmetrical leukoencephalopathy [8, 9]. We detected multifocal edema in the basal ganglia, capsula interna and both cerebellar hemispheres which may reflect direct toxicity of the drug or ischemic changes. Interestingly, the affected areas of the brain show highest expression of mu-opioid receptors and therefore potentially high susceptibility to opioid overstimulation [4]. In addition, the described pattern of the lesions showing restricted diffusion, no contrast enhancement and additional affection of deep gray nuclei and corpus callosum have been linked to toxic and metabolic disorders [10] and go beyond the typical presentation of acute toxic leukoencephalopathy [11]. Considering the possible differential diagnosis for toxic or metabolic disorders (e.g. carbon monoxide (CO), hepatic/hyperammonemic encephalopathy, hypoxic-ischemic encephalopathy (HIE), osmotic demyelination syndrome (extra-pontine myelinolysis) and the clinical features and history of the patient, methadone/opiate-induced encephalopathy along with hypoxic-ischemic encephalopathy are the two most probable causes for the described injury.

As the intravenous methadone consumption was only performed at a single point of time, one would expect a decrease in symptoms correlating with the damage seen in radiological imaging as well as CSF. Controversially, this case shows an improvement of clinical findings yet new lesions in the crura cerebri along with a shift in monocyte subtypes and a decrease in the CD56bright NK-cell-fraction in the control exams at day 11. Other cases confirm this clinical course of improved symptoms along with the increasing extent of reduced diffusion imaging after opiate-consumption [11]. However, even though others previously showed a correlation between opiate-related acute toxic leukoencephalopathy and elevation of CSF myelin basic protein suggesting myelin damage [12], the current case report may be the first to present a CSF leukocyte analysis suggesting a secondary inflammatory process.

Our hypothesis of a secondary inflammatory damage is based on surprisingly specific abnormalities in CSF cell composition characterized by a shift of monocytes from classical (CD14 + CD16-) to non-classical (CD14 + CD16+) and shift from CD56bright non killer (NK) cells to CD56dim NK cells. Notably, both CD14 + CD16 + -monocytes and CD56dim NK cells are known pro-inflammatory cell types which may play a role in the delayed encephalopathy [13, 14]. To further support the hypothesis of secondary inflammatory damage against delayed toxic effects one could take a determination of myelin basic protein into consideration, as it has been shown that myelin basic protein can be elevated in acute toxic leukoencephalopathy [12].

We observed persistent neurocognitive impairments at day 18, especially in tests for cognitive processing speed, verbal and visual learning capacity as well as verbal memory recall. This pattern is in line with previous studies reporting a positive association of larger cognitive impairment with higher methadone doses (for a review see [5]). Associations between domain-specific cognitive impairments and local of brain lesions are however highly variable in young patients due to their usually high levels of cognitive reserve and related compensatory mechanisms [15].

In summary, intravenous methadone intoxication may lead to both primary metabolic-toxic as well as diffuse CNS toxicity that could be interpreted as secondary delayed and potentially inflammatory encephalopathy with persistent neurological deficits including neurocognitive impairment.

## Supplementary Information

**Additional file 1: Supplementary Figure 1.** Additional MRI imaging on day 1 and day 11. Timepoint 1 (Day 1 after intoxication): A: DWI sequence above the latter ventricle; B: FLAIR sequence above the latter ventricle Timepoint 2 (Day 11 after intoxication) FLAIR sequences: C: DWI sequence above the latter ventricle; D: FLAIR sequence above the latter ventricle.

**Additional file 2: Supplementary Table S1.** Raw Scores and z-scores of neuropsychological test results.

## Data Availability

Not applicable.

## Abbreviations

ADC: Apparent diffusion coefficient
BVMT-R: Brief visuospatial memory test-revised
CD: Cluster of differentiation
CNS: Central nervous system
CSF: Cerebrospinal fluid
dB: Decibel
D-KEFS: Delis-Kaplan executive function test
ECG: Electrocardiogram
EEG: Electroencephalogram
FACS: Fluorescence-activated cell scanning
FAST: Focused assessment with sonography for trauma
FLAIR: Fluid attenuated inversion recovery
MRI: Magnetic resonance imaging
NK: Non killer
RAVLT: Rey auditory verbal learning test
SD: Standard deviation
SDMT: Symbol digit modalities test
TAP: Test of attentional performance
TMT: Trail marking test
TOF: Time-of-flight
