# Supplementary figures and images for: Intravenous methadone causes acute toxic and delayed inflammatory encephalopathy with persistent neurocognitive impairments
Source: BMC Neurol. 2021 Feb 22;21:85. doi: 10.1186/s12883-021-02108-9 (PMC7898738; doi:10.1186/s12883-021-02108-9)

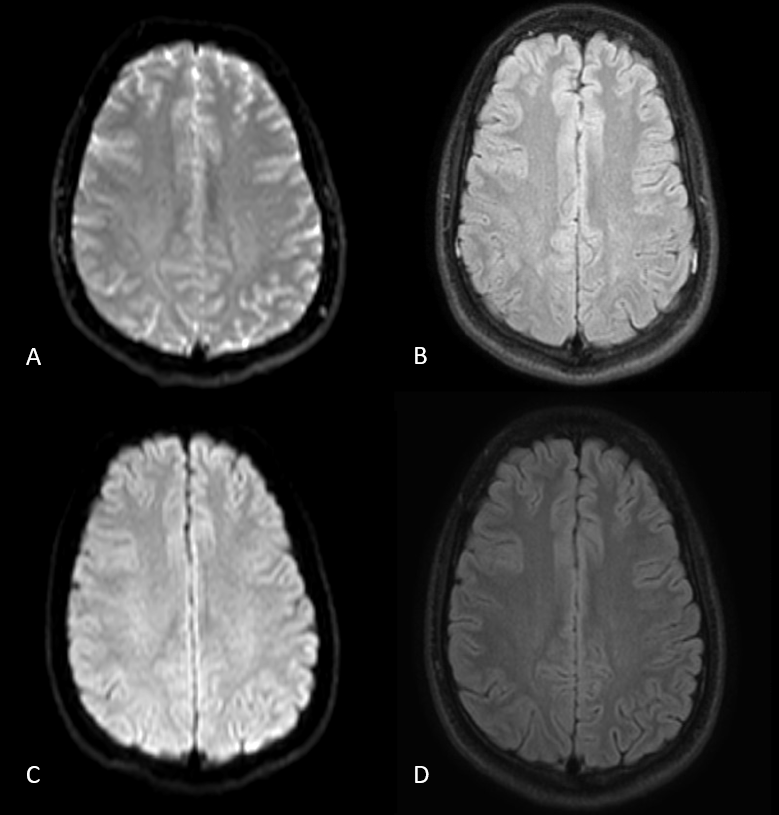

Supplement: Supplementary file 1 — Additional file 1: Supplementary Figure 1. Additional MRI imaging on day 1 and day 11. Timepoint 1 (Day 1 after intoxication): A: DWI sequence above the latter ventricle; B: FLAIR sequence above the latter ventricle Timepoint 2 (Day 11 after intoxication) FLAIR sequences: C: DWI sequence above the latter ventricle; D: FLAIR sequence above the latter ventricle. [file 12883_2021_2108_MOESM1_ESM.png]
