# Supplementary material for: Intravenous methadone causes acute toxic and delayed inflammatory encephalopathy with persistent neurocognitive impairments
Source: BMC Neurol. 2021 Feb 22;21:85. doi: 10.1186/s12883-021-02108-9 (PMC7898738; doi:10.1186/s12883-021-02108-9)
Supplement: Supplementary file 2 — Additional file 2: Supplementary Table S1. Raw Scores and z-scores of neuropsychological test results. [file 12883_2021_2108_MOESM2_ESM.docx]

| Supplementary Table S1. Raw Scores and z-scores of neuropsychological test results. | | |
| --- | --- | --- |
| **Cognitive Domain** | **raw score** | **z-score** |
| **Global Cognitive Screenings** |  |  |
| MoCA | 29 | .10* |
| **Verbal Learning** |  |  |
| RAVLT 1 | 7 | -.17 |
| RAVLT 5 | 13 | -.26 |
| RAVLT 1-5 | 49 | -.92 |
| **Verbal Memory** |  |  |
| RAVLT 6 | 10 | -1.07 |
| RAVLT 7 | 10 | -.95 |
| RAVLT W-F | 13 | -.59 |
| **Visual Learning** |  |  |
| BVMT-R 1 | 3 | -2.20 |
| BVMT-R 3 | 12 | .78 |
| BVMT-R 1-3 | 23 | -1.33 |
| **Visual Memory** |  |  |
| BVMT-R 4 | 12 | .88 |
| BVMT-R % Retained | 100 | .71 |
| **Visuoconstruction** |  |  |
| RCFT Copy | 35 | -.06 |
| **Cognitive Processing Speed** |  |  |
| SDMT (oral) | 47 | -1.91 |
| TMT A (sec.) | 27 | -.59 |
| RCFT time (sec.) | 210 | .48 |
| Tonic Alertness (Median, sec.) | 258 | -1.2 |
| Phasic Alertness (Median, sec.) | 242 | -0.8 |
| **Attention % Executive Functions** |  |  |
| Digit span forwards | 9 | 1.00 |
| Digit span backwards | 7 | .07 |
| TMT B (sec.) | 46 | .23 |
| 5 Point Test (correct) | 38 | .99 |
| 5 Point Test (flexibility) | 2.63 | .10 |
| TMT B/A | 1.70 | .77 |
| D-KEFS Tower Test Achievement Score | 24 | 1.70 |
| D-KEFS Tower Test Time per move ratio | 3.57 | -.3 |
| Note. MoCA, Montreal cognitive assessment; RAVLT, Rey auditory verbal learning test; BVMT-R, Brief visuospatial memory test-revised; RCFT, Rey complex figure test; SDMT, symbol digit modalities test; TMT A, Trail making test part A; TMT B, Trail making test part B; D-KEFS, Delis-Kaplan Executive Function System; *normative data for age groups >=65. | | |
